# The Dual Use of the Pyranine (HPTS) Fluorescent Probe: A Ground-State pH Indicator and an Excited-State Proton Transfer Probe

**DOI:** 10.1021/acs.accounts.2c00458

**Published:** 2022-09-02

**Authors:** Ramesh Nandi, Nadav Amdursky

**Affiliations:** Schulich Faculty of Chemistry, Technion − Israel Institute of Technology, Haifa 3200003, Israel

## Abstract

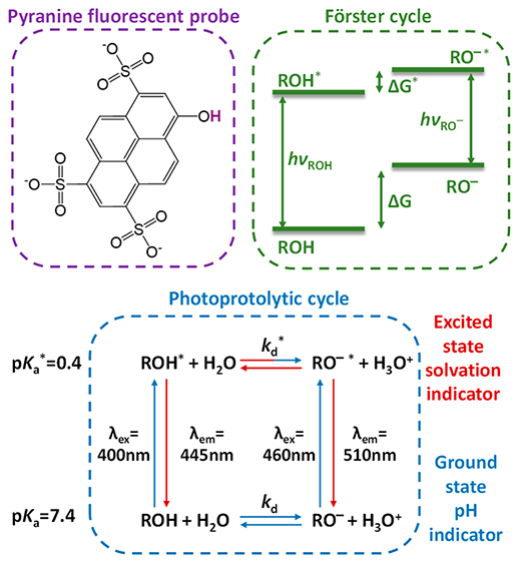

Molecular fluorescent probes are an essential experimental tool in many fields, ranging from biology to chemistry and materials science, to study the localization and other environmental properties surrounding the fluorescent probe. Thousands of different molecular fluorescent probes can be grouped into different families according to their photophysical properties. This Account focuses on a unique class of fluorescent probes that distinguishes itself from all other probes. This class is termed photoacids, which are molecules exhibiting a change in their acid–base transition between the ground and excited states, resulting in a large change in their p*K*_a_ values between these two states, which is thermodynamically described using the Förster cycle. While there are many different photoacids, we focus only on pyranine, which is the most used photoacid, with p*K*_a_ values of ∼7.4 and ∼0.4 for its ground and excited states, respectively. Such a difference between the p*K*_a_ values is the basis for the dual use of the pyranine fluorescent probe. Furthermore, the protonated and deprotonated states of pyranine absorb and emit at different wavelengths, making it easy to focus on a specific state. Pyranine has been used for decades as a fluorescent pH indicator for physiological pH values, which is based on its acid–base equilibrium in the ground state. While the unique excited-state proton transfer (ESPT) properties of photoacids have been explored for more than a half-century, it is only recently that photoacids and especially pyranine have been used as fluorescent probes for the local environment of the probe, especially the hydration layer surrounding it and related proton diffusion properties. Such use of photoacids is based on their capability for ESPT from the photoacid to a nearby proton acceptor, which is usually, but not necessarily, water. In this Account, we detail the photophysical properties of pyranine, distinguishing between the processes in the ground state and the ones in the excited state. We further review the different utilization of pyranine for probing different properties of the environment. Our main perspective is on the emerging use of the ESPT process for deciphering the hydration layer around the probe and other parameters related to proton diffusion taking place while the molecule is in the excited state, focusing primarily on bio-related materials. Special attention is given to how to perform the experiments and, most importantly, how to interpret their results. We also briefly discuss the breadth of possibilities in making pyranine derivatives and the use of pyranine for controlling dynamic reactions.

## Key References

- AmdurskyN.Photoacids as a new fluorescence tool for tracking structural transitions of proteins: following the concentration-induced transition of bovine serum albumin. Phys. Chem. Chem. Phys.2015, 17, 32023–320322657399010.1039/c5cp05548b.^1^*In this paper, we show how pyranine can be used as an excited-state probe for following structural transitions of proteins.*
- AmdurskyN.; LinY.; AhoN.; GroenhofG.Exploring fast proton transfer events associated with lateral proton diffusion on the surface of membranes. Proc. Natl. Acad. Sci. U. S. A.2019, 116, 2443–24513067927410.1073/pnas.1812351116PMC6377470.^2^*In this paper, we show the possibility of chemically functionalizing the pyranine molecule to enable its tight tethering to biological membranes. In this way, the new pyranine can be used as a fluorescent probe for proton diffusion on the surface of the membrane.*
- NandiR.; YucknovskyA.; MazoM. M.; AmdurskyN.Exploring the inner environment of protein hydrogels with fluorescence spectroscopy toward understanding their drug delivery capabilities. J. Mater. Chem. B2020, 8, 6964–69743250087710.1039/d0tb00818d.^3^*In this paper, we show the capability of pyranine to serve as an excited-state probe for exploring the inner hydration environment of a protein-based hydrogel.*
- Burnstine-TownleyA.; MondalS.; AgamY.; NandiR.; AmdurskyN.Light-modulated cationic and anionic transport across protein biopolymers. Angew. Chem., Int. Ed.2021, 60, 24676–2468510.1002/anie.20211102434492153.^4^*In this paper, we show the difference in the excited-state properties of pyranine between chemisorption to physisorption of the probe to a protein material. We further utilize pyranine as light-triggered source of protons to influence protonic transport on the surface of the protein material.*

## Introduction

Fluorescent molecular probes are an essential tool in biological, biomedical, and materials studies. They can be used as solvated molecules or tethered to a certain molecule/surface. In terms of their fluorescence, we should distinguish between probes that are always “turned on”, which are always fluorescent with a specific emission peak, and fluorescent probes that change their fluorescence upon binding to a molecule or in different environments. Fluorescent probes that are always turned on are usually bound to a molecule of interest and used for the localization of it, such as fluorescent proteins. For probes that change their photophysical properties, we can distinguish between two classes. The first class includes probes that are turned on only after binding to a certain molecular surface and are used to detect the presence and localization of that surface. There are several turn-on mechanisms within this class. For instance, the common thioflavin-T probe, used to stain amyloid fibrils, is turned on due to the inhibition of its nonradiative twisted intramolecular charge transfer process after binding,^5^ while other probes, including some of the biological membrane probes, can turn on following a change in their surrounding polarity, such as going from an aqueous solution to a more hydrophobic environment.^6^ While the turn on mechanism results in a significant increase in fluorescence intensity, it is not a quantitative approach: the number of photons reaching the detector is dependent on many other parameters. The second class includes probes whose photophysical properties change upon a change in their environment.^7^ Usually, it is manifested in a shift in the emission band position and/or the ratio of two different peaks, which can be quantitative and correlated to a specific environmental feature such as the pH. Indeed, most fluorescent probes that are being used as pH indicators have two absorption/emission peaks, and the ratio between them is used to estimate the pH. A member of this class is pyranine (8-hydroxypyrene-1,3,6-trisulfonic acid, HPTS), which is the topic of this Account. We discuss the use of pyranine as a ground-state pH indicator, followed by the emergent use of pyranine as an excited-state probe for the environmentally related hydration state next to the probe, which is based on the excited-state proton transfer (ESPT) properties of pyranine.^8^

## Introduction to the Photophysical Properties of Pyranine

**Figure 1:**
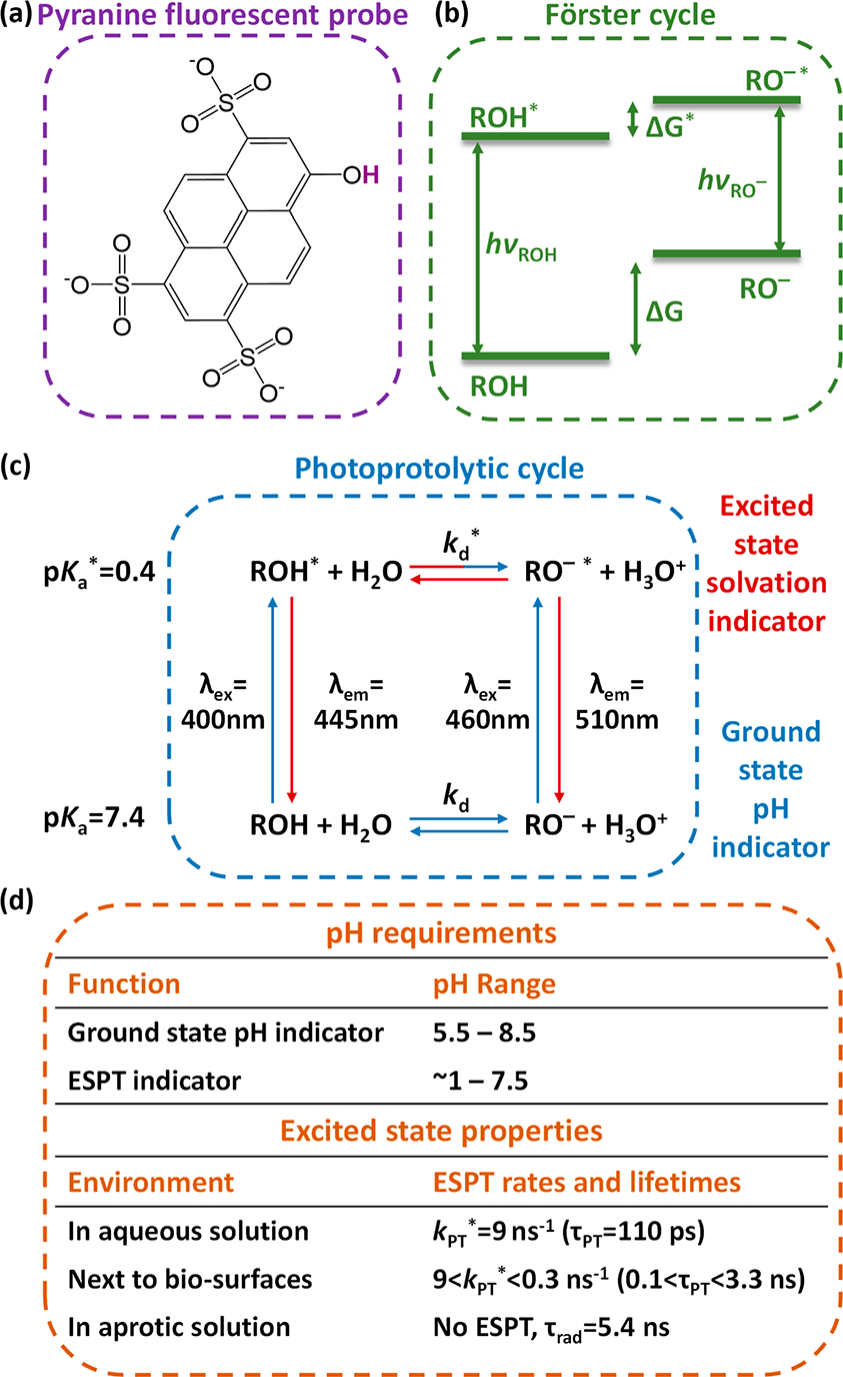
(a) Chemical structure, (b) Förster cycle, and (c) photoprotolytic cycle of pyranine. (d) Summary of the important requirements and excited-state properties for the use of pyranine as a probe (including the ESPT rate (*k*_PT_^*^) and lifetime (*τ*_PT_) and the pure radiative lifetime (*τ*_rad_).

Pyranine (Figure 1a) is an −OH-containing arylsulfonate, which can be protonated (ROH) or deprotonated (RO^–^) (ROH* and RO^–^* in the excited state). The photophysical properties of pyranine were studied decades ago independently by Ireland and Wyatt^9^ and Förster^10^ as part of a grand exploration of the excited-state acidity of many aryls. The Förster cycle (Figure 1b) describes the energy transitions between ROH and RO^–^, meaning the acid–base equilibrium, and their different properties in the ground and excited states. The S_0_ → S_1_ transition energy varies between ROH and RO^–^, resulting in different absorption and emission wavelengths for the two states of pyranine. The absorption (excitation) and emission peak positions of the ROH state are ∼400 and ∼445 nm, respectively, whereas the positions for RO^–^ are ∼460 and ∼510 nm, respectively. Furthermore, the Gibbs free energy (Δ*G*) of the acid–base transition ROH ⇄ RO^–^ + H^+^ is different for the ground and excited states, with Δ*G* < ΔG*. This change in the acid–base transition between the ground and excited states results in a large Δp*K*_a_ value between these two states, where the ground-state p*K*_a_ is 7.3–7.7 and the excited-state p*K*_a_* is 0.4–1.3. *This difference between the pK_a_values of the ground and excited states is the basis for the dual use of the pyranine fluorescent probe*. We can look at the acid–base transition of pyranine, which is called a photoacid, in a similar manner to any Brönsted–Lowry acid, meaning that in addition to pyranine as the proton donor, we need a proton acceptor, and in most cases, the acceptor is water. The photoprotolytic cycle of pyranine together with water as the proton acceptor (Figure 1c) highlights the routes relevant to the different uses of pyranine as a fluorescent probe. It is important to mention that there are many studied photoacids with large Δp*K*_a_ values and ESPT processes similar to those of pyranine, mainly naphthol-based and hydroxycoumarin-based photoacids.^11−13^ However, pyranine is by far the most used fluorescent probe of this family because (1) its absorption and emission band positions are in the visible region, (2) a large Stokes shift makes the detection easier, (3) it is highly water-soluble and negatively charged over a wide range of pH values due to its sulfonates, and (4) its p*K*_a_ value is around physiological pH.

## Pyranine as a Ground-State Fluorescent pH Indicator

### Photophysical Origin

**Figure 2:**
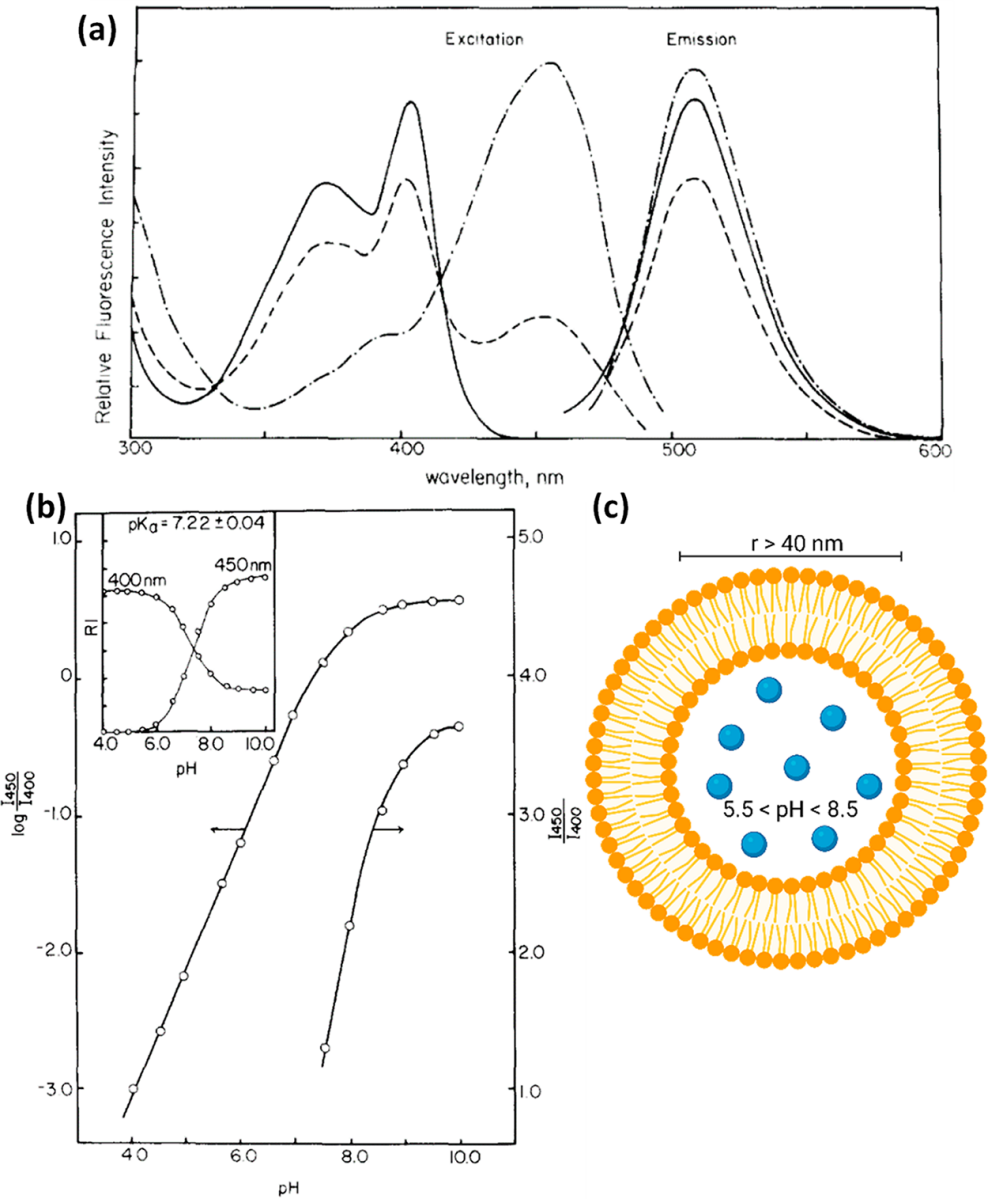
(a) Excitation (λ_em_ = 510 nm) and emission (λ_ex_ = 400 nm) spectra of pyranine in aqueous solutions at pH 4 (solid), pH 7 (dashed), and pH 10 (dot-dashed). (b) Ratio of relative intensities (*I*_450nm_/*I*_400nm_) in the excitation spectra (λ_em_ = 510 nm) of pyranine as a function of pH. The inset shows the titration curves of pyranine in the same solutions. (c) Schematic of the use of pyranine (cyan spheres) within liposomes. Reproduced with permission from ref (14). Copyright 1978 Elsevier.

**Figure 3:**
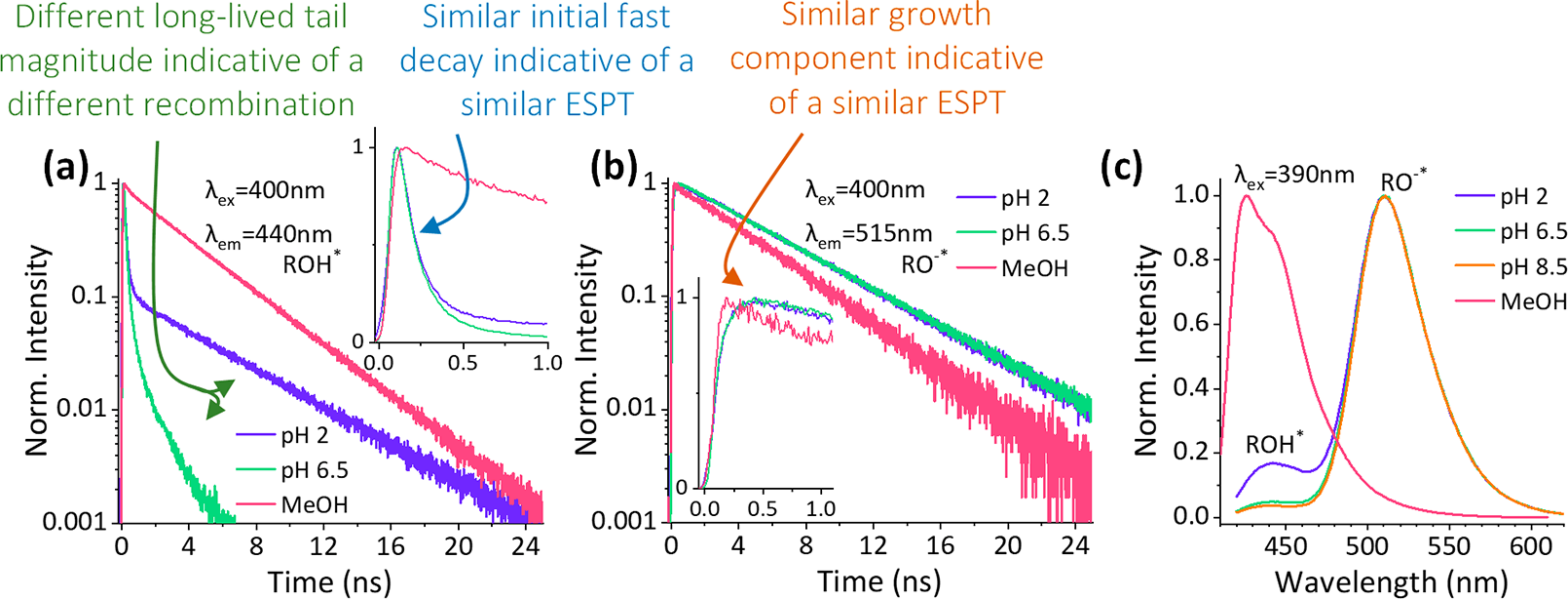
Time-resolved fluorescence decay of (a) ROH and (b) RO^–^ (the insets show the first nanosecond of the decay) and (c) steady-state fluorescence spectra of pyranine at different pH values and in methanol.

The first common use of pyranine is as a pH indicator (blue arrows in Figure 1c).^14−21^ This use is related to the ground-state p*K*_a_ of pyranine, and accordingly, it can detect the range of pH = 5.5–8.5 (Figure 1d). Importantly, at such pH values, the steady-state emission band is always that of RO^–^* at ∼510 nm (as shown also in Figures 2a and 3c). At solution pH greater than p*K*_a_, the majority of pyranine will be RO^–^, and upon excitation, we will receive the emission band of RO^–^* at ∼510 nm. At solution pH lower than p*K*_a_, the majority of pyranine will be ROH, and upon excitation it will go to ROH*. However, because of the ESPT process, ROH* will be converted into RO^–^* with emission at ∼510 nm. Hence, the steady-state *excitation* spectra at pH values above and below the p*K*_a_ are different (Figure 2a), which is due to the different routes reaching RO^–^* at these pH values. At pH > p*K*_a_, the emission of RO^–^* originates mainly from RO^–^, while at pH < p*K*_a_, the emission of RO^–^* originates mainly from ROH. Accordingly, when using pyranine as a fluorescent pH indicator, we can either follow the emission of RO^–^* upon excitation of RO^–^, with higher pH leading to higher emission intensity, or measure the fluorescence excitation spectrum at λ_em_ of RO^–^* and record the ROH/RO^–^ excitation peak ratio. As discussed above, the emission intensity is not quantitative, and to make it quantitative a calibration curve is essential. In contrast, the latter methodology based on the ROH/RO^–^ ratio in the excitation spectrum is quantitative and can be directly translated to the pH (Figure 2b), and thus, it is the recommended one.

### Use of Pyranine as a Ground-State pH Indicator

The main rationale for using soluble (and membrane-impermeable) pyranine as a fluorescent pH indicator is to evaluate the pH of confined spaces. Accordingly, the first seminal work on pyranine (in 1978) was directed to pH sensing of the aqueous phase within liposomes (Figure 2c).^14^ From this point, and taking into account the simplicity of using pyranine as a wide-range pH probe (Figure 2b), pyranine became one of the golden probes for measuring the pH within biomolecular compartments, such as liposomes, vesicles, and different types of cells.^15−21^ Nevertheless, it is worth mentioning that the known calibration curve of pyranine (Figure 2b) is dependent on the p*K*_a_ value of the probe. As shown in several studies,^22,23^ the p*K*_a_ of pyranine is sensitive to the ionic strength and the solvent surrounding it, and thus, minor changes in the calibration curve are expected for different environments.

Importantly, since the use of pyranine as a ground-state pH indicator relies on the ESPT process, it is essential that pyranine must be solvated in solution and not bound to a surface. From a visionary perspective, the mentioned first seminal paper on the use of pyranine as a pH indicator ended with the following sentences:^14^ “The present work has demonstrated the advantages of pyranine as a pH probe in liposomes. This technique can be extended to the measurement of hydrogen ion concentrations at enzyme active sites and at hydrophilic protein surfaces. The only requirement is knowledge of the location of the probe”, which brings us to the next section of this account.

## Pyranine as an Excited-State Fluorescent Probe for Hydration and Proton Diffusion

### Photophysical Origin

The second use of pyranine is related to the excited-state properties upon excitation of ROH while following the emission of ROH* and RO^–^* (dark-red arrows in Figure 1c). This use is based on the acid dissociation only in the excited-state, meaning at pH values of p*K*_a_* < pH < p*K*_a_ (Figure 1d). Over this wide pH range (∼1 < pH < 7.5), upon the excitation of ROH (∼400 nm), it can undergo an ESPT process and transfer its proton to a nearby proton acceptor, such as water. Accordingly, the mentioned acid dissociation constant in the excited-state (*k*_d_^*^) is commonly called an ESPT rate constant (*k*_PT_^*^). While we refer to *k*_PT_^*^ as the first step in the ESPT process, faster processes happen on the femtosecond time scale involving electron density redistribution and solvation,^24^ which are the basis for the photoacidity of photoacids.^25^ Both theoretical and experimental efforts have focused on the different steps in the excited state, whereas the common convention is that the ESPT results in the formation of an ion pair between the proton and the negative RO^–^*, which is followed by dissociation of the ion pair.^26−28^ In his last paper (which was published after he passed away), Dan Huppert also discussed the role of the negatively charged sulfonates in the formation of the ion pair.^29^ Nevertheless, we cannot exclude the “simpler” mechanism without this extra step, as shown in Figure 1c. In terms of time scales, the ESPT process is fast, and for pyranine in water, the ESPT has a lifetime of ∼110 ps (*k*_PT_^*^ = 9 × 10^9^ s^–1^) (Figure 1d). This fast ESPT process can be observed using time-resolved fluorescence (Figure 3a) by the rapid decay of the ROH* signal (∼440 nm) during the first 0.5 ns after excitation (the larger *k*_PT_^*^ is, the faster is the initial decay) and as a growth component of the time-resolved trace of the RO^–^* fluorescence (Figure 3b). Although the ESPT rate, quantum yield, emission/absorption peak positions, and lifetime of pyranine are very much medium-dependent,^23^ here we focus on the use of pyranine only in aqueous solutions and upon binding to (bio)surfaces. Importantly, the ESPT process can happen only if a proton acceptor is available nearby, which can be facilitated also through a chain of water molecules to an acceptor base.^30−34^ As will be discussed, this is a fundamental part of using pyranine as an indicator for the surrounding hydration layer. If we follow the fluorescence decay of pyranine in an environment that does not support ESPT, such as a solvent that acts as a bad proton acceptor (e.g., methanol), pyranine will not undergo ESPT, the ROH* will have an exponential radiative decay with a lifetime of ∼5.4 ns (Figure 3a), and the steady-state spectrum will show only the ROH* band (Figure 3c). Following the ESPT process, there is a chance for a geminate recombination process of the dissociated proton with RO^–^* to reform ROH* with rate constant *k*_a_^*^.^34−42^ This proton recombination process is highly sensitive to the proton diffusion constant and dimensionality around the photoacid.^43^ The re-formation of ROH* at longer time scales due to proton recombination is evident in the long-lived tail at the emission position of ROH*, with more efficient proton recombination resulting in a higher amplitude of the tail component. Intuitively, a higher concentration of protons in solution leads to a higher probability of a recombination process (high *k*_a_^*^). Accordingly, while comparing the decay of pyranine at low pH versus physiological pH, we can observe that the initial decay of ROH*, representing the process of ESPT from pyranine to water molecules, is similar for the two pH values, whereas the long-lived tail is very different and more prominent at low pH (Figure 3a), also resulting in a larger normalized ROH band in the steady-state measurement (Figure 3c).

### Modeling the Excited-State Dynamics of Photoacids

**Figure 4:**
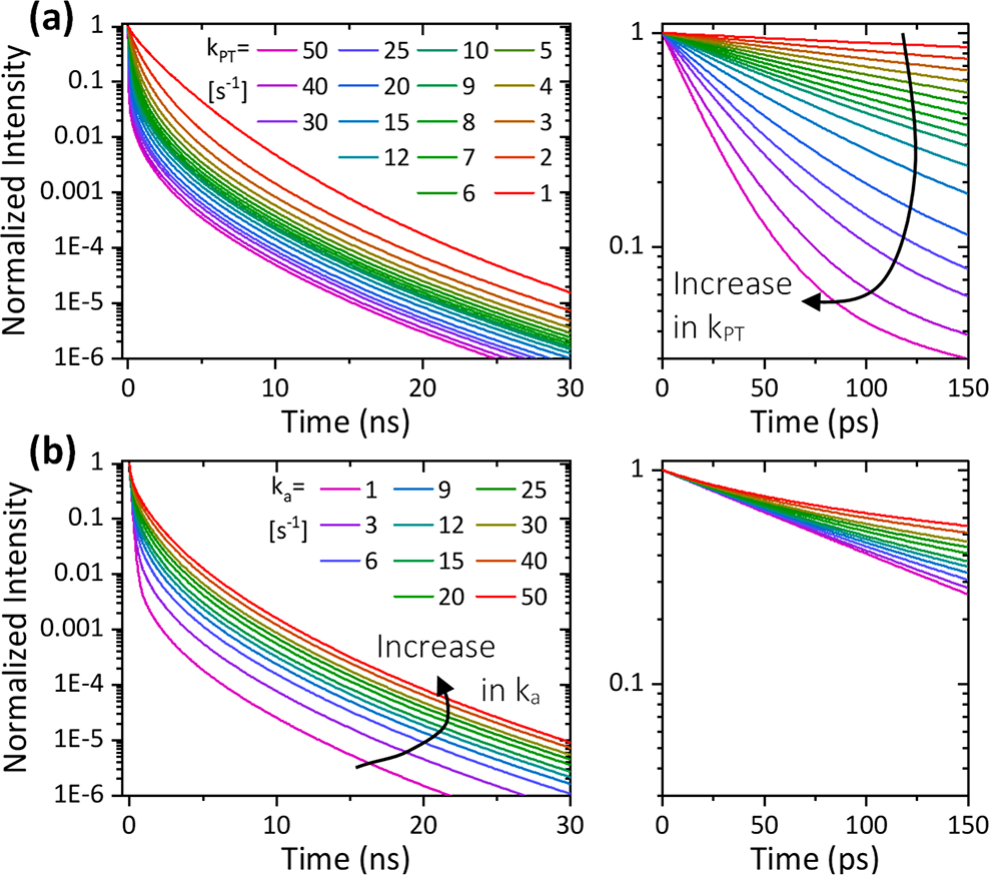
Constructed time-resolved transients of *I*_f_^ROH*^ using the SSDP software^44^ (a) at different *k*_PT_^*^ values (*k*_a_^*^ = 9 ns^–1^) and (b) at different *k*_a_^*^ values (*k*_PT_^*^ = 9 ns^–1^). The right panels are zoomed-in views of the first 150 ps.

Much theoretical effort has been employed to understand the excited-state dynamics of photoacids.^34−42^ While it is not our purpose to dwell on such theoretical effort here, we will summarize what we can learn from it. As discussed, the fluorescence transients can be used to extract *k*_PT_^*^, but other models can result in more valuable information. To date, probably the most used model is based on the work of Agmon, who used the Debye–Smoluchowski diffusion equation for the probability of the process, *P*(*t*), in a spherical symmetric diffusion problem (SSDP).^35−37,44^ The summary of the model is as follows:
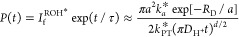
1This model follows the transient of ROH* (*I*_f_^ROH*^) corrected for the radiative lifetime (τ) and describes a process within a reactive sphere of radius *a* (containing the photoacid), where *d* is the dimensionality of the diffusion having a proton diffusion constant of *D*_H^+^_. *R*_D_ is the Debye radius, which is the distance at which the Coulombic attraction between the negative excited-state anion (RO^–^*) and the positive proton equals the thermal energy (*k*_B_*T*). For pyranine in an aqueous solution, *R*_D_ = 28 Å. The use of this model further allows us to examine the role of each component on the *I*_f_^ROH*^ transients, which is experimentally impossible, using the software developed by Krissinel’ and Agmon.^44^ For instance, we can clearly observe the change of *I*_f_^ROH*^ as a function of *k*_PT_^*^ and *k*_a_^*^, with larger *k*_PT_^*^ resulting in faster initial decay (Figure 4a) and larger *k*_a_^*^ leading to slower decay, which is more prominent at longer time scales (Figure 4b).

### The Importance of Time-Resolved Fluorescence Measurements

In the first part of this Account, concerning the use of pyranine as a ground-state pH indicator, we discussed how a “simple” steady-state fluorescence excitation spectrum is needed in order to use pyranine. On the contrary, to use pyranine as an excited-state fluorescent probe, we claim that steady-state fluorescence measurements are not enough and that there is a need for a time-resolved measurement. Steady-state measurements represent average information after infinite time, and as discussed, the different processes in the excited-state of the photoacid and their associated parameters are at different time scales following excitation. Accordingly, a high-intensity ROH* emission peak in steady-state measurements can be a result of poor *k*_PT_^*^, efficient *k*_a_^*^, reduced proton diffusion dimensionality, or small diffusion coefficient. Thus, only by time-resolved measurements we can differentiate among different processes. Interpretation of the time-resolved decay of pyranine is the basis for the use of pyranine as an excited-state probe, which will be described in the next section. Importantly, as shown in Figures 3a and 4 (and the related text), the decay of ROH* can provide valuable information concerning both the ESPT and recombination processes. Accordingly, in all of the examples below, we show only the ROH* decay (at ∼440 nm).

### Use of Pyranine as an Indicator for Confined Water and as a Distance Ruler

**Figure 5:**
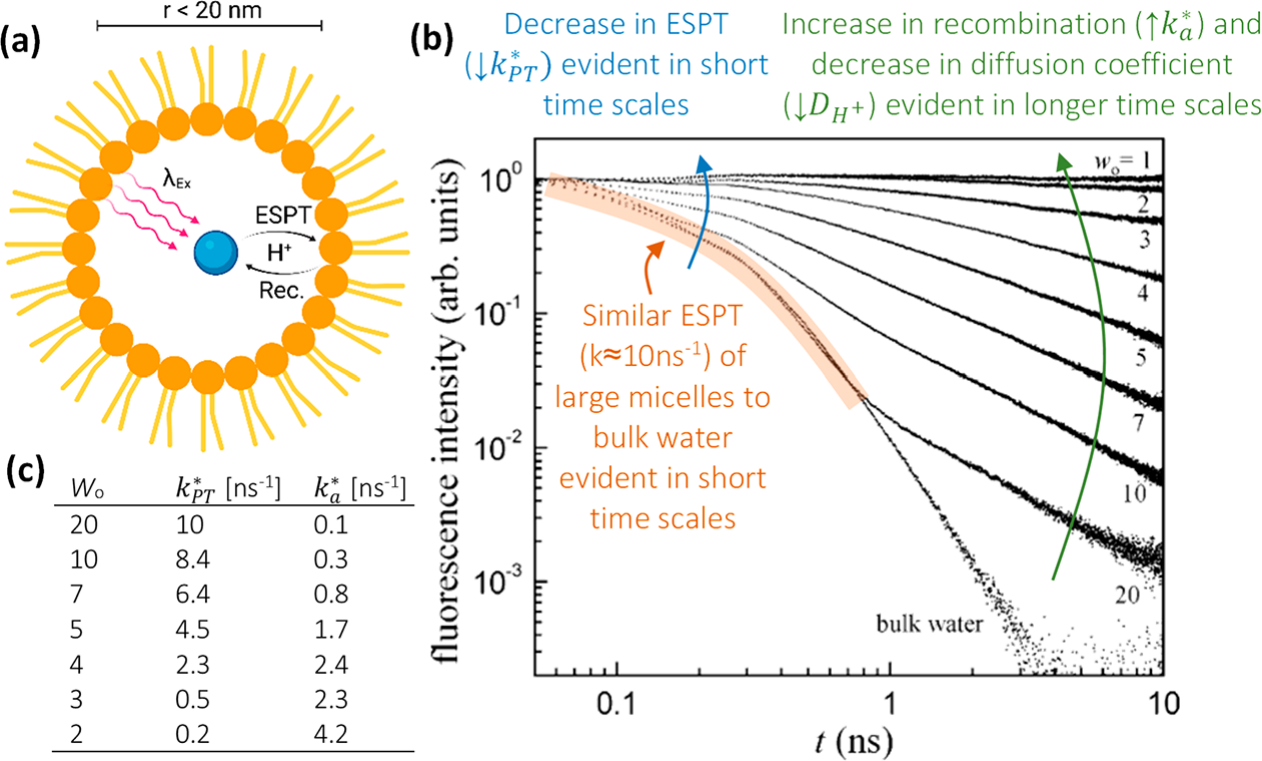
(a) Schematic of the use of pyranine (cyan spheres) within reverse micelles, (b) fluorescence decay (on a log–log plot) of pyranine within them (where *w*_0_ is the mole ratio of water to surfactant molecules), and (c) the *k*_PT_^*^ and *k*_a_^*^ values extracted using an exponential fit of the decay. Reproduced from ref (45). Copyright 2007 American Chemical Society.

One of the first uses of pyranine as an excited-state probe was to follow the ESPT in a confined space, i.e., a nanopool (Figure 5a). Such an environment is very different from what we discussed in the first part, where pyranine was solvated within liposomes, in which the inner aqueous partition within the vesicle is treated as bulk solution. Here, the extremely low water volume creates a unique environment (water shells) different than bulk water, inhibiting the ESPT from pyranine, restricting proton diffusion following ESPT, and promoting efficient geminate recombination. Accordingly, the smaller the size of the nanopool (i.e., the smaller the volume of water in the confined space), the poorer are the ESPT efficiency (smaller *k*_PT_^*^) and diffusion ability and the more efficient is proton recombination, resulting in considerably slower decay of ROH* (Figure 5b,c). In such a way, the transient of pyranine can be correlated with the size of the confined space. Although this property is not a direct distance measurement, such as in FRET measurements, we can still refer to it as an indirect distance ruler. Nevertheless, we should remember that we are after the excited-state processes, which are limited to a few dozens of nanoseconds, thus highly limiting the proton diffusion that can be observed to the nanometer scale. Taking this limiting condition, the maximum diameter of the confined space (the nanopool) that can be followed and distinguished from bulk water is ∼20 nm, which corresponds to the diffusion length of the dissociated proton during the excited-state lifetime of pyranine. Due to the necessity of having solvated pyranine (or other photoacids) in such a small volume, this use of pyranine was mainly targeted to study reverse micelles (as in Figure 5) and water-containing porous membranes or hydrogels.^45−52^ It is also important to mention that any association between pyranine and the surfactant/material results in major changes in the transients.^53−55^

### Use of Pyranine as an Indicator for Local Hydration and Proton Diffusion

The use of pyranine as a ground-state pH indicator relies on the solvation of pyranine in different aqueous environments. However, in terms of the unique excited-state properties of solvated pyranine in aqueous environments, this environment is less interesting. As discussed above, no matter what the pH of the solution is, the ESPT efficiency will be rather similar, and the only difference is the proton recombination rate in the excited state. To probe the local hydration surrounding the pyranine molecule and to follow proton diffusion to/from it, the probe should be placed at a specific location that exhibits different hydration properties than bulk aqueous solution. In the following sections, we will discuss specific biological interfaces. Importantly, while most of the studies target water as the proton acceptor for pyranine, in some cases placing pyranine next to a biological material results in other moieties/bases serving as proton acceptors.

#### Within Binding Sites of Proteins

**Figure 6:**
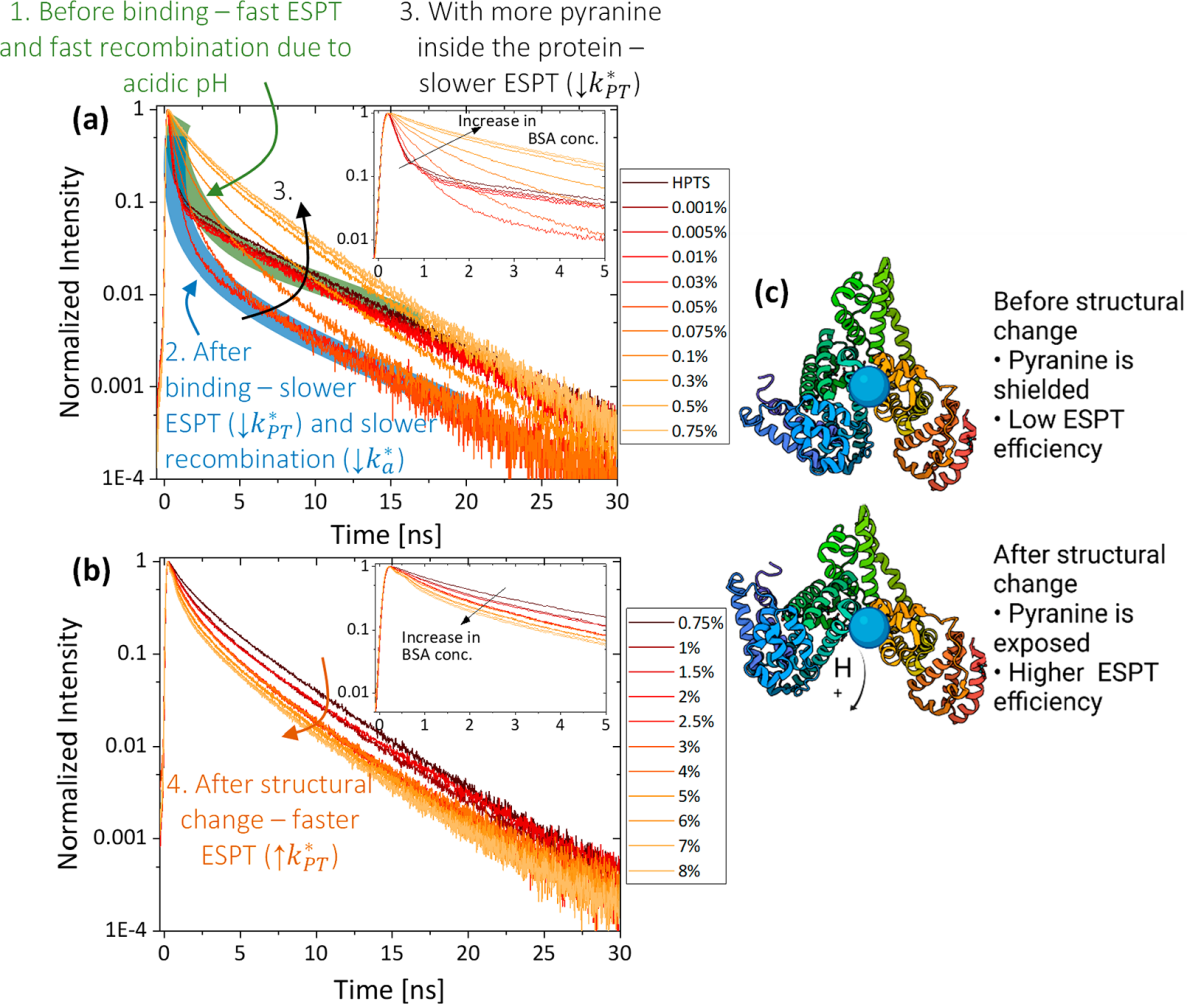
Time-resolved emission of pyranine in BSA fractions of (a) ≤ 0.75% and (b) ≥ 0.75%. The insets show magnifications of the first nanoseconds. (c) Schematic of the system. Reproduced with permission from ref (1). Copyright 2015 The PCCP Owner Societies.

Usually, a binding site within a protein is shielded to some extent from the bulk solution via the protein envelope. Indeed, the binding of pyranine to a binding site of the serum albumin protein resulted in a poorer ESPT efficiency compared to water,^1,56,57^ indicating the hydrophobic environment of the binding site and the restricted motion of water molecules within the protein. This ability of pyranine to sense the accessibility of water to binding sites can be used to follow any structural change of the binding site. It was shown that bovine serum albumin (BSA) undergoes structural changes as a function of concentration at low pH values, resulting in exposure of the binding site to the bulk solution and thus markedly influencing the ESPT efficiency of the bound pyranine (Figure 6).^1^ The figure shows the change in the ROH* transient upon binding to the protein, resulting in slower ESPT up to the point of the structural change (from *k*_PT_^*^ ≈ 5 ns^–1^ before binding to 0.4 ns^–1^ after binding), after which the ESPT becomes faster (increasing to *k*_PT_^*^ ≈ 1 ns^–1^), whereas the values were extracted using exponential fitting of the initial decay. While the binding of pyranine at binding sites usually restricts the ESPT, there is the possibility that an additional proton acceptor is present in such sites, specifically, glutamic and aspartic acid residues. In one of the studies, such alternative ESPT resulted in even more efficient ESPT compared to pyranine in solution.^58^

#### Next to the Surface of Protein-Based Macropolymers

**Figure 7:**
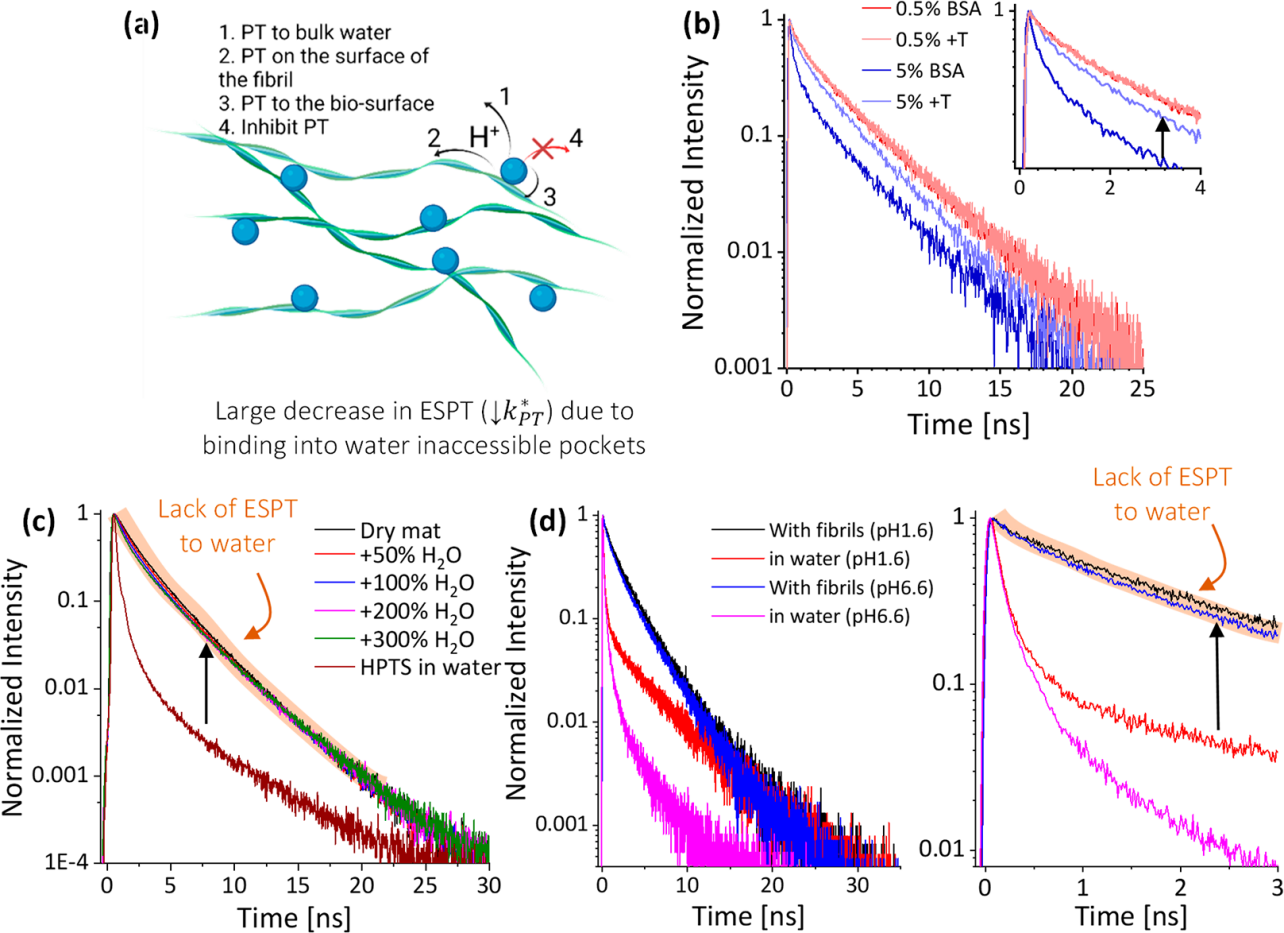
(a) Schematic of pyranine on the surface of fibrils. (b–d) Time-resolved emission of pyranine (b) on the surface of BSA before and after thermal treatment for the 0.5% and 5% BSA samples, where only the 5% samples undergo gelation; (c) on the surface of protein mats at different wt % of water compared to pyranine in bulk water; and (d) on the surface of amyloid fibrils at different pH values. Panel (b) is reproduced with permission from ref (3). Copyright 2020 Royal Society of Chemistry. Panel (c) is from ref (59). CC BY 4.0. Panel (d) is reproduced from ref (61). Copyright 2014 American Chemical Society.

Whereas above we discussed probing the binding sites of solvated proteins, here we focus on probing the water environment of macroscopic biostructures. Such studies rely on adsorbing pyranine to the surface of the bio-structure, such as hydrogels, mats, films, or fibrils (Figure 7a). Thus, using pyranine allows both the study of the inner hydration layer of such surfaces and deciphering of the proton diffusion along the biological surface. For instance, BSA can undergo thermally induced gelation, but only above a certain protein concentration.^3^ Using pyranine, it was shown that the surface of the protein becomes more hydrophobic after gelation (Figure 7b), as indicated by significantly slower ESPT after gelation (*k*_PT_^*^ ≈ 3.5 → 0.9 ns^–1^), resulting in an insight into the gelation mechanism.^3^ In other cases using protein mats,^59^ protons released on the surface of the mat did not diffuse into bulk water, as adding more water did not induce a change in the fluorescence transient (Figure 7c). Importantly, the binding mode to the biological surface is of prime importance, where it was shown that chemisorption (covalent binding) of pyranine to a protein mat resulted in an increase in ESPT of pyranine compared to physisorption of pyranine to the same protein mat.^4^ This was explained via ESPT from the covalently attached pyranine to nearby carboxylates of the protein, which are less accessible using physisorption of pyranine. Another important protein-based system is amyloid fibrils, where in line with the above-mentioned fibrils, also here the ESPT was significantly slower on the surface of the amyloid fibril compared to bulk water, indicating the poor water accessibility of the surface next to the binding site of pyranine and the lack of ESPT to bulk water, regardless of the pH of the solution (Figure 7d).^60,61^

#### Within Chains of Polysaccharides

**Figure 8:**
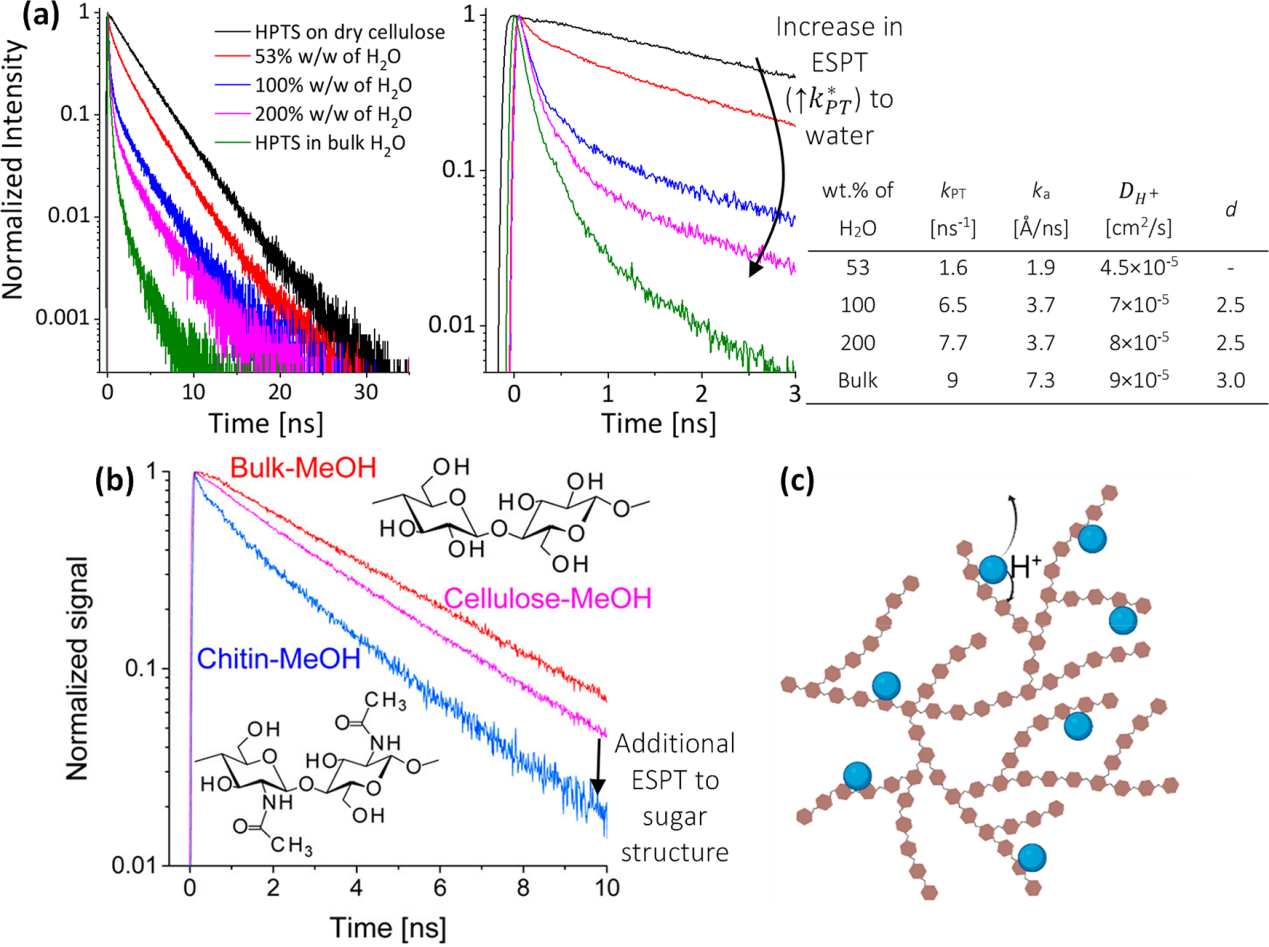
(a, b) Time-resolved emission of pyranine (a) adsorbed on cellulose in the dry state for several weight percentages of H_2_O added (relative to cellulose), compared to pyranine in bulk water together with the extracted parameters according to eq 1, and (b) adsorbed on cellulose and chitin in a methanolic solution, compared to pyranine in bulk methanol. (c) Schematic of the experimental systems showing the two possibilities for ESPT. Reproduced from refs (61) and (63). Copyright 2014 and 2015, respectively, American Chemical Society.

An additional bio-related environment that was probed using pyranine is the aqueous phase within polysaccharides, both linear and branched, including cellulose, chitosan, and starch samples.^61−63^ Polysaccharides are hydrophilic, and the goal of such studies was to explore the water state within them. Intuitively, the ESPT of pyranine within polysaccharides is highly dependent on their water content (Figure 8a), and it was shown that ESPT is faster when more water is added to the material and that the polysaccharide strands limit the dimensionality of proton diffusion. An interesting example of a polysaccharide surface exhibiting different properties is chitin (acetylated polysaccharide),^63^ in which the weakly basic acetyl groups can serve as proton acceptors, resulting in faster ESPT at a very low water content and even in the presence of significant amounts of methanol and ethanol (Figure 8b).

#### On the Surface of Biological Membranes

**Figure 9:**
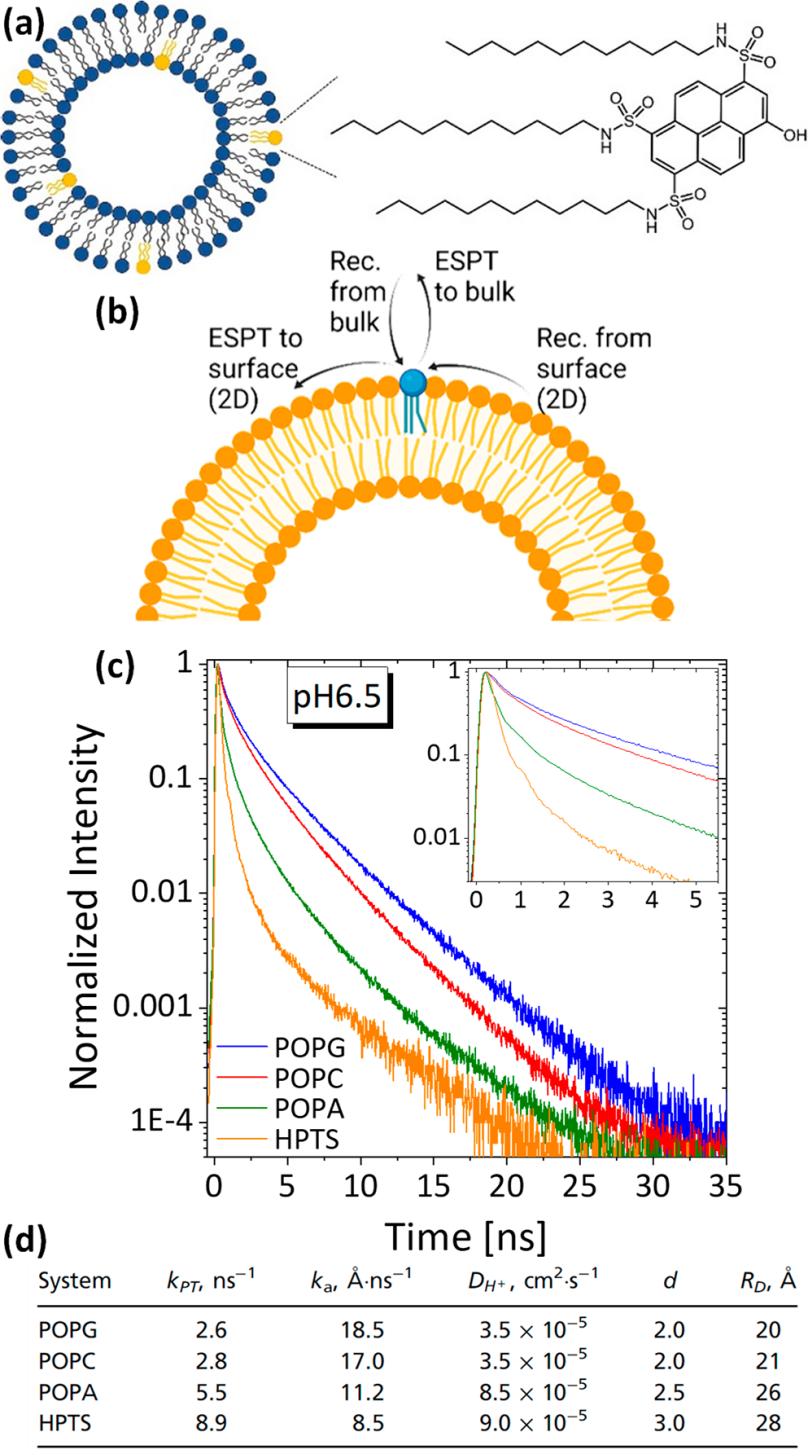
(a) Molecular structure of C_12_-HPTS. (b) Schematic of the ESPT processes involving the surface of the membrane. (c) Time-resolved emission of pyranine in several lipid vesicles at pH 6.5. (d) Extracted parameters according to eq 1. Reproduced from ref (2).

Above we discussed cases where pyranine was absorbed into the biological materials. This strategy relies on electrostatic or hydrophobic interactions between the sulfonates or the pyrene moiety and the biological surface in question. However, in some cases this is not enough, and there is a need to place pyranine in a specific location that requires chemical modification of pyranine. While discussing biological membranes, it was shown that adding long 12-carbon alkyl chains to the sulfonates of pyranine (termed C_12_-HPTS) allowed the modified pyranine to be tethered into lipid membranes (Figure 9a).^2^ In this way, the proton diffusion on the surface of the membrane or surface-to-bulk PT (Figure 9b) was explored, and it was shown that the lipid headgroup strongly influences the fluorescence transient (Figure 9c). Such transients were used with the above-described model to extract the different ESPT efficiency and proton diffusion parameters (Figure 9d). It was concluded that some phospholipids can support lateral proton diffusion with limited bulk-to-surface proton transfer, whereas others are more accessible to protons in solution and permit proton diffusion from the surface to the solution. This study also showed the profound effect of the pyranine environment on its ground- and excited-state properties, whereas the same probe (C_12_-HPTS) exhibited a major shift in the p*K*_a_ values from 9.8 in anionic lipids to 3.2 in cationic ones, thus limiting its use as an excited-state probe in cationic lipids only to very low pH values. The ability of C_12_-HPTS to serve as a fluorescent sensor for proton diffusion also allowed it to be used as a sensor for detecting any minimal membrane perturbations.^64^ In this context, we should also mention earlier studies in which pyranine was used to study the ability of membranes to serve as proton-collecting antennas that captured protons released from nearby solvated pyranine molecules.^65^

### Limitations and Requirements for the Use of Pyranine as an Excited-State Probe

**Figure 10:**
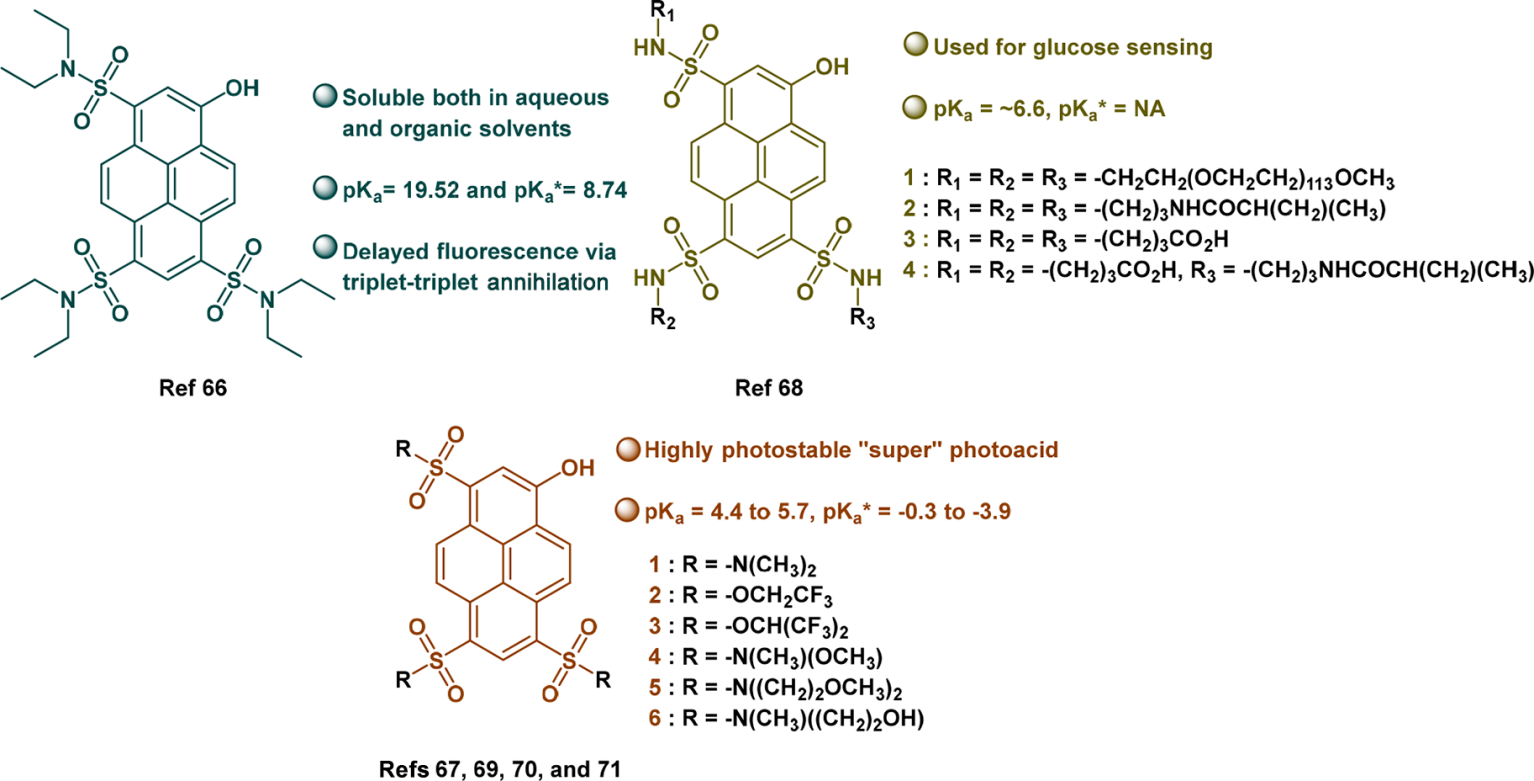
Various reported derivatives of pyranine photoacids and their unique features.^66−71^

As discussed, the use of pyranine as an excited-state probe can result in fundamental new understandings related to the ability of pyranine to undergo ESPT, followed by proton diffusion and recombination taking place in the excited-state. Accordingly, we must excite the pyranine in its ROH state to utilize it as an excited-state probe. This can be a major limitation of pyranine (or any other photoacid) that limits its use under alkaline (high-pH) conditions and in some cases (as discussed for the cationic lipids) only to acidic conditions. Hence, the p*K*_a_ of pyranine in a given location must be measured before experiments. In this context, it should be noted that the p*K*_a_ and p*K*_a_* are also sensitive to the ionic strength of the environment. Two more drawbacks of pyranine are its relatively high rate of bleaching compared to other photoacids, which can be ascribed to its three sulfonic acid groups, and its smaller Δp*K*_a_ compared to other naphthol-based photoacids. To address the mentioned limitations, there is a need to target the sulfonic acid groups of pyranine to make new derivatives, and a variety of chemical functionalizations with different substituents of pyranine have been achieved (Figure 10).^66−71^ Importantly, we do not discuss here derivatives that targeted the −OH group, as those are not photoacids. It was shown that neutralizing pyranine by making sulfonamides can shift p*K*_a_/p*K*_a_* toward higher values.^66,67^ Such derivatives can also be utilized specifically to target saccharide sensing.^68^ An additional class of derivatives decreased the p*K*_a_* of pyranine to values as low as −3.9 and were termed *super photoacids*.^69−71^ Such super photoacids undergo a significant red shift into the visible region and show improved stability, further increasing the attractiveness of their use, specifically for imaging applications.^71^ On the other side, the very large ESPT rate (*k*_PT_^*^) of super photoacids can be also a drawback due to the experimental difficulty of deciphering it.

## Future Perspective for the Use of Pyranine

The main focus of this Account is on the emerging use of pyranine as an excited-state probe for the local hydration and proton diffusion properties of a (bio)surface. In this application, there is a need to bind pyranine to a specific site. One approach to do so is via electrostatic interactions between pyranine and the probed surface. Another approach is via chemical derivatizations of pyranine. As discussed, such derivatizations can result in the integration of pyranine into specific sites. We predict that halide derivatization of the sulfonate groups will be an attractive avenue, as it will allow covalent binding of the modified pyranine to various nucleophiles via S_N_2 nucleophilic attack,^4,72−74^ as biosurfaces have a variety of nucleophiles (e.g., amines, carboxylates, or polar groups). On the other hand, click chemistry is also an attractive envisioned derivatization of pyranine that would allow a more specific functionalization of an azide-functionalized pyranine to an alkyne-functionalized surface. While in this Account we have mostly focused on the sensing of biologically relevant environments, it is important to mention that similar methodologies have been employed to sense the surfaces of nonbiological hard and soft materials via covalent functionalization of the material with pyranine.^73−76^ An additional exciting avenue for the utilization of pyranine is not for (bio)sensing but rather for light-triggered release of the proton, which already started from two of the earliest studies on pyranine showing laser-induced pH jumps of concentrated pyranine solutions.^77^ In this way, the on-demand and very fast (subnanosecond) formation of a proton next to a specific location can dynamically influence pH-sensitive processes^72,78−82^ or even can serve as a protonic charge carrier in proton-conductive materials.^4,83−87^

## Dedication

This Account is dedicated to the late Prof. Dan Huppert from Tel Aviv University, who taught me (N.A.) everything I know on this topic.
